# Building freeways: piloting communication skills in additional languages to health service personnel in Cape Town, South Africa

**DOI:** 10.1186/s12913-017-2313-1

**Published:** 2017-06-07

**Authors:** Joel Claassen, Zukile Jama, Nayna Manga, Minnie Lewis, Derek Hellenberg

**Affiliations:** University of Cape Town, Cape Town, South Africa

**Keywords:** South Africa, Language learning, Career-orientated language teaching, Health care barriers, Multilingualism, Health care staff, Afrikaans and isiXhosa

## Abstract

**Background:**

This study reflects on the development and teaching of communication skills courses in additional national languages to health care staff within two primary health care facilities in Cape Town, South Africa. These courses were aimed at addressing the language disparities that recent research has identified globally between patients and health care staff. Communication skills courses were offered to staff at two Metropolitan District Health Services clinics to strengthen patient access to health care services. This study reflects on the communicative proficiency in the additional languages that were offered to health care staff.

**Methods:**

A mixed-method approach was utilised during this case study with quantitative data-gathering through surveys and qualitative analysis of assessment results. The language profiles of the respective communities were assessed through data obtained from the South African National census, while staff language profiles were obtained at the health care centres. Quantitative measuring, by means of a patient survey at the centres, occurred on a randomly chosen day to ascertain the language profile of the patient population. Participating staff performed assessments at different phases of the training courses to determine their skill levels by the end of the course.

**Results:**

The performances of the participating staff during the Xhosa and Afrikaans language courses were assessed, and the development of the staff communicative competencies was measured. Health care staff learning the additional languages could develop Basic or Intermediate Xhosa and Afrikaans that enables communication with patients.

**Conclusions:**

In multilingual countries such as South Africa, language has been recognised as a health care barrier preventing patients from receiving quality care. Equipping health care staff with communication skills in the additional languages, represents an attempt to bridge a vital barrier in the South African health care system. The study proves that offering communication skills courses in additional languages, begins to equip health care staff to be multilingual, that allows patients to communicate about their illnesses within their mother tongues.

## Background

In multilingual countries, language and the accompanying knowledge of culture, has been identified as a barrier preventing patients from accessing quality healthcare. Research has shown that patients whose primary language differs from that used in the health system have a poorer understanding of their diagnosis, treatment recommendations and medication than other patients, who receive treatment within a mother tongue. It is, therefore, essential that healthcare professionals can communicate and convey essential information in the patient’s mother tongue, in order to deliver the best possible service, with the best possible outcome for the patient.

Research carried out predominantly in developed countries has established that doctor-patient communication is a central clinical function in maintaining a therapeutic doctor-patient relationship as this is important in the delivery of appropriate and effective high-quality health care [1–7] Further research has shown that language discordance significantly affects access to care, causes problems of comprehension and adherence, and decreases the satisfaction and quality of care [1]. Patients with limited proficiency in the primary language of the health system are less likely to receive the care they need, their access to health is diminished and they are more likely to have a poorer understanding of the care they have received. This compromises their quality of care compared to similar patients who are able to communicate in the primary health system language. Similar situations have been reported in the developing world. In India, the language barrier has similarly been found to inhibit effective healthcare [8].

Strategies such as hiring multilingual healthcare workers, providing language training to healthcare providers, employing in situ translators or using telephone interpretation services have all proven to be effective to varying degrees in overcoming language barriers. However, these approaches are not “affordable or feasible in cash-strapped health systems” nor foolproof solutions [9]. In an ideal context, the interpreter possesses sufficient clinical, linguistic and cultural knowledge to facilitate meaningful exchange between the provider and recipient of healthcare services. However, research suggests that interpreters are often not competent enough to adequately perform the task. A study was conducted in a South African psychiatric hospital in which they examined the basic translation competencies of interpreters on site [10]. None of the participants were professionally qualified interpreters. Of the six interpreters who formed the basis of the study, two were administrative clerks, two were nurses and two were security guards. The facility’s use of these individuals seemed to be justified by the lack of available posts for professional interpreters, paired with the inability of clinicians to communicate in the patient’s language. The pressure to use untrained interpreters, in spite of the potential for negative consequences was underpinned by the argument that the transfer of information, albeit an approximation of the original intended meaning, was preferred to obtaining no information at all. An analysis of the competency levels of the interpreters in the above study revealed that the use of unsuitable individuals was not conducive to optimal care, as it led to misdiagnoses and compromised the quality of health care. By using untrained bilingual or multilingual interpreters, an assumption is often made that the ability to speak a particular language qualifies a person to transfer information from sender to recipient. A scrutiny of back-translations confirms that competence in both languages involved, and with sufficient knowledge of the translated material, will ensure closer approximations between the intended and translated meanings [10]. It also states that if and when interpreters are used, it is important that health care providers are aware of the variety of roles that interpreters can play. Some studies show that interpreters do not play a conduit role, a role where information is relayed verbatim and where the control of communication lies with the healthcare provider. Instead, interpreters often unintentionally assume the role of ‘co-diagnosticians’ [11]. Within the context of health communication, interpreters “would at times assume a specific role that enabled them to provide services typically associated with providers” which is exemplified by “(a) interpreters’ active involvement in the patients’ diagnostic and treatment process and (b) the overlapping roles and services between providers and interpreters” [11]. Setting the existing dynamics between health professionals and interpreters aside, evidence from a systematic review of the literature [12], demonstrates that the use of professional interpreters brings about more of an improvement in clinical care than when ad hoc interpreters are used. By utilising professional interpreters, studies show that there is a rise in the quality of clinical care for patients with limited English proficiency to a point which is approximate, or equal to that of patients without language barriers. However, it should also be noted that these approaches are not universally accepted.

Monroe and Shirazian [12] assert that some commonly employed methods of interpretation are potentially dangerous. The authors acknowledge the findings of previous studies on the use of untrained interpreters which stress that an inadequate transfer of vital information may give rise to misdiagnosis, or even result in fatal consequences. Karliner et al. [11] caution that the use of interpreters in medical consultations may lead to errors of omission about frequency and duration of treatment, allergies and side effects caused by drug dosages. Relevant data in the patient history and miscommunication leading to the volunteering of incorrect information both on the side of the physician and on that of the patient is also noted, resulting in poor outcomes.

The extensive use of such strategies is ubiquitous within the public health care system of South Africa. The country’s healthcare system faces many challenges, which include but are not limited to, a shortage of skills and equipment, difficulties with staff retention and the challenge of providing an efficient and equitable service to patients from diverse cultural and linguistic backgrounds. These population dynamics are experienced by staff at those hospitals which attract many patients who struggle to communicate with the predominantly English speaking medical staff [12]. Many staff members at health facilities in the Western Cape are bilingual (and speak either English and Afrikaans, or isiXhosa and English). This makes the provision of healthcare within the patient’s home language challenging, especially at the primary health care level with potential adverse effects on patient access to healthcare, the quality of care that these patients receive which [13] with potential adverse effects on patient access to healthcare, the quality of care that these patients receive which would enable them to continue with their own healthcare at home. Furthermore, communities have become increasingly multicultural and ethnically heterogeneous and there is a need to be alert to the diverse challenges this may bring.

In South Africa, as in other multilingual countries, more than 80% of doctor-patient consultations take place across both language and cultural barriers. Demeurt [14] and Crawford [15] have recognized the need to bridge this communication barrier. Several studies (amongst others [15–17]) have investigated the impact of language barriers on the client and health care provider relationship and confirm that patients prefer being addressed in their own language and that satisfaction is higher when they are consulted in their own mother tongue. The second-biggest obstacle for clients to access health care services, after income, is health care professionals not being able to communicate in their patients’ language. Good communication has long been acknowledged as the cornerstone of the health professional-patient relationship and plays an important role in the quality of health care delivery, at the heart of effective diagnosis and treatment lies the medical interaction between physician and patient.

Key studies that confirm how the difficulties with communication and cultural incompatibility are important barriers to quality health care were conducted at the suburban Red Cross War Memorial Children’s Hospital (Cape Town, South Africa) [18], while other studies established that although patients cited socio-economic issues as barriers to optimal care, communication with patients was stressed, as a significant barrier to health care [14, 17]. The manner in which the language barrier undermines effective health care provision at Madwaleni Hospital in the rural Eastern Cape confirms how the language barrier decreases work efficiency and the provision of holistic treatment [19–21]. Additionally, it makes communication time-consuming which increases frustration levels and decreases empathy and approachability.

Thus, language alone appears to be a barrier to the attainment of quality healthcare. It has adverse effects on patients and is seen as a barrier to the patients’ understanding of their doctors’ instructions. Karliner, L.S., et al. [11] describes the language practices at three hospitals in the Western Cape where the focus was on communicating with isiXhosa-speaking patients, as this is an area where the language barrier is most strongly felt and inequalities are most extensive. The research revealed that patients whose primary language differed from that used in the health system, had a poorer understanding of their diagnosis, treatment recommendations and medication than other patients. Even though English is often the *lingua franca* (common language) by which communication occurs at the health centres with patients, miscommunication is clearly a common occurrence within South African facilities that needs to be eliminated as a daily stumbling block to healthcare [12, 13, 20, 22].

In the South African context, the absence of professional interpreters at sites of health care provision is the norm due to a variety of factors, including those of a financial and logistical nature. A solution should therefore be sought which complements the existing health system in order to improve the quality of health care for those with limited proficiency in the language in which it is provided. Therefore, this paper will reflect on evaluating the effectiveness of equipping healthcare providers with communication skills in career-orientated isiXhosa and Afrikaans that revolve around the healthcare setting between providers and patients.

Ideally doctors should therefore speak their patients’ languages, but compelling doctors to learn their patients’ language may neither appear practical or achievable. An approximation of this ideal can be attempted on the level of doctor training. The proposal is that learning the patient’s language should be seen as one step on the journey to relative language competence. The advantage of this initiative is that competent doctors can take full responsibility for mediating meaning during consultations and have the assurance that the intended medical advice reaches the client. The on-going challenge would, however, be to also develop the ability to fully comprehend patient utterances within the relevant cultural context of each client.

Following on [20] promoting the primary health care approach, there was an international movement for medical curricular reform to meet the knowledge, skills and values requirements of medical graduates as stated in the declaration. The Health Professions Council of South Africa (HPCSA) promulgated guidelines for the education and training of doctors in South Africa in a discussion document in February 1999. As part of its curricular reform, the University introduced isiXhosa and Afrikaans into the new MBChB curriculum in 2003 as part of the Becoming-a-doctor course in the Division of Public Health and Family Medicine. It has contributed to empowering medical students, interns and doctors with work-based, career-orientated Afrikaans and isiXhosa since its inception. The challenge of providing communication skills training to primary health care facilities staff remains, and this project aimed to begin redressing this problem.

Pilot career-orientated communication skills courses for Beginners in isiXhosa and Afrikaans for health provider personnel, accredited by UCT’s Centre For Higher Education (CHED), were offered at the Delft and Kraaifontein Community Health Centres (CHC’s). The study identified three key factors that should be considered in addressing the problem, namely:

- ways in which language barriers affect health and healthcare,
- efficacy of interventions to overcome language barriers and
- costs of language barriers and efforts to overcome them.

The need to address language barriers in health worker education and clinical practice through language programmes at educational institutions and access to language learning resources was also identified [8].

Through this initiative, the Research team and the Northern Tygerberg Sub-structure (NTSS) of the Western Cape Department of Health, have attempted to evaluate the impact these communicative language courses have had on existing staff at the two CHC’s.

### Objectives

The objectives of the pilot course were to:

- Offer a twelve week course in career-orientated communication skills at the healthcare facilities where the language disparities between staff and patients were determined to be the largest.
- Determine and describe the language disparities between the home languages of patients and staff at the Kraaifontein and Delft CHCs (in the NTSS of the MDHS).
- Measure the language proficiency of health professional staff members participating in the project prior to, during and subsequent to the language training.
- Conduct course evaluations at the conclusion of each round.
- Develop the pilot course into a formal course for health care providers in the Western Cape province.

The aim of the pilot project was to address the language barrier at the two healthcare facilities and improve the access of isiXhosa and Afrikaans-speaking patients to healthcare services, by improving the healthcare provider and patient communication, accepting the challenge of [23] that: “The relationship between a health-care professional (HCP) and his/her client requires communication, but meaningful communication is only possible through a language that is intelligible to both the interlocutors”.

## Methods

### Course material development

The curricula for the basic language training for CHC staff members were developed for the primary healthcare setting through cooperation between the health care facility staff at the two Northern Tygerberg Substructure clinics, staff of the Division of Family Medicine, and the African Languages and Afrikaans and Netherlandic studies sections.

An initial curriculum was presented to the NTSS management team by the project team to ascertain which topics would be relevant within the clinical setting of the CHC’s. The proposed course material was derived from the isiXhosa and Afrikaans career-orientated courses that had been offered within the Becoming-a-doctor undergraduate courses in the Faculty of Health Sciences and was extracted or adapted from courses such as Becoming a Doctor Part 2A and Part 2B and Afrikaans for Beginners. The material incorporated from the MBChB-curricula at UCT needed to be adapted to meet the communicative needs of the participant healthcare workers at the CHC’s who have had very little communicative ability in isiXhosa and Afrikaans. Management and participating staff thus had an opportunity to give critical input into a final draft curriculum. In this manner, the relevance of the existing student curricula could be critically interrogated, as the immediate day-to-day communicative needs of staff at the community health centres needed to be taken into consideration to determine the range of topics to be covered as opposed to the university students who would not encounter this broader-base of topics until they graduated. The healthcare workers, on the other hand, need to interact with patients regarding various critical topics ranging from history taking, relaying diagnostic information, instructing patients, the use of medication and adherence to same.

The language courses offered at the Delft and Kraaifontein CHC’s were offered at an entry-level for the learning of foreign languages [Common European Framework; Interagency Language Roundtable scale (ILR) Level 0, or American Council on the Teaching of Foreign Languages (ACTFL) Novice level or Defense Language Proficiency Test (DLPT) level 0]. The participants had varying low levels of competency in the respective languages and this had to be considered when developing the material in order to facilitate effective learning for all participants. However, given the positive feedback from participants and the pass-rate at the various CHC’s it is evident that the chosen material exceeded the needs and expectations of those who participated in the courses offered.

Two rounds of entry-level languages courses were offered for each of the two languages, while one Intermediate-level course was offered for each language. Each round of the communication skills courses allowed for the enrolment of up to 15 participants per language to ensure that the facilitator could provide successful support within the class context. Clinical staff were primarily targeted for participation, however administrative staff subsequently comprised up to 50% of the class participants. The challenge was that the communication skills material derived from the medical school communication skills curricula, for specific use by the healthcare workers, would then need to be amended for other participants, who are performing administrative functions within the facilities. The language facilitators, however, opted to keep the core teaching material focussed on the needs of the healthcare staff, while ancillary material was not developed for staff, but their needs were rather addressed within classes where generalisations related to clinic functions, could benefit both the healthcare staff and the administrative staff.

Language facilitators also left the beginners and intermediate course curriculum open to comment from the participating staff, to ensure that the presented content remained vital and relevant. For this very reason, topics that were considered too theoretical or less useful within the clinical setting were not edited in the communication skills manuals utilised by the healthcare workers in Round 2.

The study involved mixed methods, with a quantitative focus in evaluating the language profile of the Western Cape populace, the language profiles of the health care staff members and the performances of the staff learning the communication skills.

## Results

### Provincial language profile

**Fig. 1:**
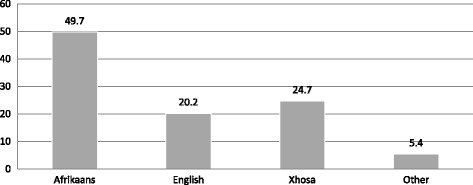
Language profile of Western Cape residents in Census 2011

In preparation for offering the courses the language profiles of the community health centres and the neighbourhoods, in which they are located, had to be considered. Delft and Kraaifontein are heterogeneous sub-economic suburbs in the Northern suburbs of the Cape metropole, populated by indigenous Afrikaans and Xhosa-speaking inhabitants of Cape Town, as well as foreign and domestic inhabitants who have relocated. The South African national census of 2011 reported that 94.5% of Western Cape residents are speakers either of Afrikaans, English or IsiXhosa [24] (Fig. 1).

The NTSS management staff of the Provincial Government of the Western Cape proposed that the pilot study be conducted at the Delft and Kraaifontein Community Health centres based on personnel language data. However, the project ran an independent quantitative study to determine the language disparities between the healthcare providers and the patients.

### Patient language profiles

To confirm the language profiles, a multilingual interviewer interviewed over 100 patients randomly chosen at the Delft and Kraaifontein sites respectively (Table 1). The questionnaires were completed by patients at the waiting-rooms at the healthcare facilities who randomly elected to complete the questionnaire during January and February 2015. The result of the language questionnaire to patients confirmed that Afrikaans was the predominant language spoken by 53% of patients surveyed at the Delft CHC, while 34% of the surveyed patients spoke isiXhosa. IsiXhosa was the predominant language spoken at the Kraaifontein CHC (47%), while Afrikaans (37%) and English (6%) were the other prominent languages in use amongst the patients surveyed at Delft. English was found to be the third-most prevalent language, spoken as a mother tongue by 10% of the surveyed patients. The disparity between staff and patients that utilize English as a home language will be highlighted as it is acutely evident. The staff members’ language profiles were provided by the Northern Tygerberg substructure (NTSS). The findings of the patient survey thus measure up equitably to the polling of languages to the 2011 National census reported above. Upon comparison, the language profile of each CHC reveals that the language ratio between the healthcare staff and the patients measures up in the following manner (Table 2).

**Table 1:** Home languages of Patients attending Delft and Kraaifontein CHCs

Healthcare Facilities (CHC)	Number Patients by Home language				
	Afrikaans	English	isiXhosa	Other	Total
Delft	56	11	36	3	106
	52.83%	10.38%	33.96%	2.83%	100%
Kraaifontein	40	6	50	11	107
	37.38%	5.61%	46.73%	10.28%	100%
Total	96	17	86	14	213
	45.07%	7.98%	40.38%	6.57%	100%

**Table 2:** Comparison of Staff and Patient Language Profiles

Healthcare Facilities (CHC)	Number of Staff by Home Language							
	Afrikaans	English	French	North-Sothu	Urdu	isiXhosa	isiZulu	Total
Delft	45	89		1	1	29	1	166
	27.11%	53.61%	0	0.60%	0.60%	17.47%	0.60%	100%
Kraaifontein	49	60	1			11	1	122
	40.16%	49.18%	1%	0	0	9.02%	0.82%	100%
Total	94	149	1	1	1	40	2	288
	32.64%	51.74%	0.35%	0.35%	0.35%	13.89%	0.69%	100%

*N* = 288

This table stresses that the language profiles of the staff members and patients at the respective CHC’s are significantly different. What is of immense concern is that more staff members at both CHC’s speak English as a home language, 54 and 49% at Delft and Kraaifontein, respectively, compared to only the 10 and 6% of the respective patient populations.

English is the first or second additional language of 73% of patients surveyed at the two facilities and the fact that the day-to-day practice is that patients are interviewed, evaluated and treated in English by staff, as it is the *lingua franca* between the healthcare practitioner and patient. This emphasizes the barrier that language poses in the healthcare sector and that this study has intended to address.

## Discussion

### Multilingualism amongst patients

Given the heterogeneous composition of South African sub-economic communities, as opposed to recent South African history, one would expect a measure of multilingualism within the communities. The language profile patient survey revealed the extent of multilingualism amongst surveyed patients as summarized below (Table 3).

**Table 3:** Multilingual patients per Language at Delft and Kraaifontein

Home language	Number of Patients able to communicate in at least 1 additional language		
	Yes	No	Total
Afrikaans	82	14	96
	85.42%	14.58%	100%
English	16	0	16
	100%	0%	100%
isiXhosa	68	18	86
	79.07%	20.93%	100%
Other	14	0	14
	100%	0%	100%
Total	180	32	212
	84.91%	15.09%	100%

Of the surveyed patients, 85% reported that they could communicate in at least one additional language, other than their mother tongue. This finding reflects on the practice of multilingual patients, capable of code-switching, who alternate between communicating in their home language and then switching to health care provider’s language to communicate, to compensate for the health care provider’s inability to communicate in the patient’s mother tongue. This is a practice that millions of South African healthcare practitioners rely on, but it could also be to the detriment of the individual patient’s medical diagnosis, treatment and progress. The percentage of the surveyed Afrikaans-speaking patients (L1) who reported being multilingual is 85%, while 79% of Xhosa-speaking patients indicated that they were multilingual. The English-speaking patients (L1) who were surveyed on that particular day at the two CHC’s all reported that they were not multilingual, or did not consider themselves multilingual.

### Staff feedback

Many staff members consider themselves to be relatively multilingual. The critical issue around staff skills needs was explored by the following staff questionnaire (Table 4).

**Table 4:** Staff perceptions of own language needs

Questions	Staff responses to questions			
1. How often do you have to speak Afrikaans/isiXhosa with patients?	Daily 48%	Often 18%	Seldom 14%	Incomplete 20%
2. Do you think it is important for the staff at the clinic to speak in the patient’s language?	Yes 80%		No 0	No Answer 20%

The pilot project was designed to discern the extent of the language barrier between the healthcare worker and the patient, by means of language questionnaires for both patient and staff. The staff language questionnaire made it possible to ascertain and report on the staff members’ home languages and additional spoken languages. The course questionnaire revolved around establishing what the participants’ communication skills were, by ascertaining their own level of multilingualism and need for the additional language communication skills.

The participating CHC staff unanimously (88%) reported a need for “successful communication” between patients and healthcare staff (in the anonymous staff language questionnaire) as motivation to learn the new language of their choice.

### Language training scores

The communication skills courses to staff were a unique offering embraced by health care staff, who understood the impact it would have on their daily interaction with the clients. The participating staff members were eager to apply the communication skills to their respective departments and often class materials were accordingly supplemented [e.g. by developing materials for section such as the pharmacy, the HIV-clinic, physiotherapy, dentistry, as well as the facility reception].

Although the courses had the support of district and facility management, several impediments also existed in the offering of the courses. To conserve and respect the role course participants had to play at their facilities, the courses were offered at the health care facility sites by the communication skills lecturers. The health care staff were granted permission by the facility mangers to enroll in the communication skills as a training module. The practicalities were often difficult to contend with at the clinics, as staff had their duties to perform. The staff enrolled in the courses were adult learners and the age groups varied from staff in their late-twenties to their late-fifties.

The communication skills courses were scheduled for once a week for between nine and ten o’clock in the morning, so as to allow the staff to first attend to their respective daily tasks. However, due to the sheer volume of patients that attended each health care facility, participating staff were at times unable to attend the classes. Subsequently, the course had a high attrition rate, as staff were often unable to divest themselves from duties at the facilities. Facility management did attempt to ensure that the work of participating staff members would be covered, but often unforeseen factors such as illness, other training engagements, or district and facility training had impacted on this provision. Despite every effort to provide for an appropriate space in which the communication skills course could be offered, to utilise the teaching space due to other facility needs that used the same space (e.g. planning, other ongoing training courses, or district and facility audits].

In spite of the above factors, many staff members persisted and were however able to complete the course. It must be remembered that the participating staff members were adults, learning an additional language, with the content specifically geared to a much singular context. It is against this background, that the staff were assessed in week 6 and week 12 of the Beginners modules. Staff were not assessed in week 1, or prior to the commencement of the course, as it was feared that the staff who had volunteered could be dissuaded to continue with the course, if they perceived the level of tuition as being too difficult (Table 5).

**Table 5:** Afrikaans assessment mean scores

Class	Afrikaans Pre (*n* = 20)	Afrikaans Mid (*n* = 9)	Afrikaans End (*n* = 7)
Beginners 1	0	47.82	64.9
Beginners 2	0	39.5	62.4
Intermediate	33.9	52.4	61.4

Data from the assessments was captured in an excel spreadsheet and analysed using Stata software. The statistics in Tables 6 is not based on samples, but rather the sizes of the entire groups that participated. However, due to attrition caused by workplace stressors, by week 2 participants would drop out, and even more so by the time of the first assessments in week 6.

**Table 6:** isiXhosa assessment mean scores

Class	Xhosa Pre	Xhosa Mid	Xhosa End
Beginners 1	0	62.2	72,5
Beginners 2	0	73,05	74,8
Intermediate	29	47	63

For example, the Afrikaans beginners courses would start at the Delft community health centre (CHC) in September 2013 where the participants would be *n* = 20, but by the time of the first assessment in November 2013, *n* = 9 [standard deviation 13,79, confidence interval 44 ± 14] and by the time of the final assessment in late-January 2014, *n* = 7 [standard deviation 15,15, confidence interval 62 ± 15].

Thus also the standard confidence intervals are affected by the small statistical sample, which is in effect the entire group.

The staff performances during assessment can however be summarized as follows for each language:

The tables above reflect the mean mark obtained by classes for both the isiXhosa and Afrikaans at the two participating facilities obtained during the formative oral assessments held midway during the courses, as well as the summative assessment that concluded the course at the end of week 12. Participants confirmed that they had had no formal tuition in the languages, and were assumed to have negligible communicative ability in each language. As the pilot courses were aimed at willing staff members who had no academic training in the language they had registered for, the participants’ ability is assumed to be at the level of the Common European Framework Interagency Language Roundtable scale (ILR) Level 0, or American Council on the Teaching of Foreign Languages (ACTFL) Novice level or Defense Language Proficiency Test (DLPT) level 0. The exact assessment tool was used throughout each round of assessment, during both the formative and the summative assessment.

At the mid-point of the round 1 and round 2 the Afrikaans Beginners courses, the mean scores were 48 and 39% respectively, while by the end of the course the mean scores were 65 and 62% respectively. The mid-point mean scores of the round 1 and round 2 isiXhosa Beginners courses were 62 and 73% respectively, while by the end of the course the mean scores were 73 and 75% respectively. For the Intermediate Afrikaans module the participants initially scored a mean of 52% and by the end of the course the mean was 61%. Similarly, the initial Intermediate Xhosa mean score was 47%, while the mean score was 63% by the conclusion of the course. It is evident that for both the isiXhosa and the Afrikaans courses, that there was a marked improvement from the initial formative assessments right at the onset of the courses as well as mid-way, and the summative at the end. Participants conceivably ended courses with communication skills either at Common European Framework Interagency Language Roundtable scale (ILR) Level A2, American Council on the Teaching of Foreign Languages (ACTFL) Novice Mid 0/0+ and Defense Language Proficiency Test 0+, although this could be challenged given the short duration of the course offering.

The average obtained by the participants of the intermediate course indicates that by this stage (the end of a second round of, and more advanced level of language learning) participants had successfully mastered the basic communication skills and a more advanced level of the language. Furthermore, this information bears witness to the fact that, the participants had made a concerted effort, and a successful one at that, to transform themselves into more integrated healthcare professionals and community health centre staff members serving those from varied cultural and language backgrounds that frequent the participating CHC’s.

## Conclusion

Training doctors and healthcare staff in the language of their patients, also means that the CHC staff are being exposed to the culture of the patients. The importance of motivation in language learning, the value of being immersed in the language and culture one is learning, and the role of prior exposure to language learning, were commonly mentioned as valuable during staff feedback. The doctors deeply valued the improved rapport and deeper relationships with patients that resulted from their language learning efforts. Communicating in the language has been suggested to establish a deep relationship with patients; and remove barriers between the doctor and patient. This helped staff members to communicate at a deeper level than what was previously possible using an interpreter. Confidentiality in the doctor-patient relationship was also improved. Patients loved to speak their native language with the doctor, even if they could speak English. The effects of the language barrier were considerable and persistent despite an official language policy in the province, but by empowering staff with more communicative competence through future planned courses, the barriers to providing patients with equitable treatment can be overcome.

## Abbreviations

MDHS: Metro District Health Services
NTSS: Northern Tygerberg Substructure
PGWC: Provincial Government of the Western Cape
UCT: University of Cape Town
